# Micromechanics modelling for mineral volume fraction determination: application on a terrigenous formation

**DOI:** 10.1038/s41598-020-73775-w

**Published:** 2020-10-06

**Authors:** Rubén Nicolás-López, Jaime Meléndez-Martínez, Alfredo López-Lena-Estrada, Oscar C. Valdiviezo-Mijangos, Carlos Couder-Castañeda, Enrique Coconi-Morales, José A. España-Pinto

**Affiliations:** 1 Instituto Mexicano del Petróleo, Eje Central Lázaro Cárdenas Norte 152, Gustavo A. Madero, San Bartolo Atepehuacan, 07730 Mexico City, Mexico; 2 Instituto Politécnico Nacional, Centro de Desarrollo Aeroespacial, Belisario Domínguez 22, Centro, Cuauhtémoc, 06610 Mexico City, Mexico

**Keywords:** Solid Earth sciences, Geophysics

## Abstract

This work presents a non-linear Self-Consistent (SC) micromechanics method to model the observed physical elastic properties of a terrigenous formation with the purpose to obtain its depth mineral volume fractions profile. In this approach, it is first assumed that the observed physical elastic properties obtained from well logs, such as the density $\rho_{o}$ and the elastic compressional $Vp_{o}$ and shear $Vs_{o}$ velocities, are a non-linear relationship of the unknown mineral volume fractions $\alpha$. Then, a gradient descent algorithm is implemented to seek for those volume fractions $\alpha$ for which differences between modelled and observed physical elastic properties are minimum. It is assumed that quartz, calcite and clay are the main comprising minerals of the formation. Obtained volume fractions profile follow the same general trends to those estimated by implementing the Linear Least-Squares Inversion LLSI method which is widely used in petrophysical analysis to obtain mineral concentrations from density $\rho_{o}$, photoelectric effect $Pe_{o}$ and compressional slowness $\Delta tp_{o}$ well logs. Results also show that calcite and clay volume fractions from these two methods are highly correlated while quartz volume fractions show low correlation. Further comparison between clay concentrations from SC method with clay concentrations calculated from direct measurements of gamma ray GR well logs used as a guideline also exhibits high correlation. These results suggest that the SC method is better suited to obtain clay and calcite volume fractions rather than quartz volume fractions. However, SC method can provide with insights about the general distribution of quartz along the borehole.

## Introduction

One of the main purposes of petrophysical analysis is to obtain rock mineral volume fractions from geophysical well log data. This contribution aims to use an SC scheme to estimate mineral volume fractions from the elastic response of downhole physical properties. Obtained mineral volume fractions can be further used to assist to identify lithology along the borehole.

Methods pursuing to find mineral volume concentrations commonly assume that geophysical well logs are a linear dependence to the rock mineral volume concentrations^1–3^. At a given depth, this dependence can be stated as follows:

1 $$ {\mathbf{{\rm P}\Lambda = \Gamma }}, $$

where ${{\varvec{\Gamma}}}$ is a vector representing the well log values while ${\mathbf{\rm P}}$ is a matrix containing the corresponding known physical properties of the main pure minerals that constitute the rock.${{\varvec{\Lambda}}}$ is a vector that contains the unknown rock mineral volume fractions. Finding a solution for ${{\varvec{\Lambda}}}$ in Eq. (1) is generally carried out by using linear inversion methods, where the best estimation is found by optimizing the difference between well log data and their modelled prediction. The solution for ${{\varvec{\Lambda}}}$ in Eq. (1) using LLSI is given by

2 $$ {\mathbf{\Lambda = }}\left( {{\mathbf{\rm P}}^{{\mathbf{T}}} {\mathbf{\rm P}}} \right)^{{{\mathbf{ - 1}}}} {\mathbf{\rm P}}^{{\mathbf{T}}} {{\varvec{\Gamma}}}. $$

The main advantage of the LLSI method is that Eq. (2) can be easily solved and implemented by using any programming language. Furthermore, the linear independence between columns in ${\mathbf{\rm P}}$ guarantees the uniqueness of the solution of Eq. (2)^4^. Most common well logs used in linear inversion methods to find mineral volume concentrations include density $\rho_{o}$, sonic transient time $\Delta tp_{o}$ and photoelectric factor $Pe_{o}$. Neutron porosity and resistivity well logs can be also taken into account when fluid concentration and rock porosity are required to be quantified. Therefore, for a rock mainly composed by quartz, calcite and clay, Eq. (1) can be expressed as

3 $$ \left[ {\begin{array}{*{20}c} {\rho_{QTZ} } & {\rho_{CA} } & {\rho_{CL} } \\ {Pe_{QTZ} } & {Pe_{CA} } & {Pe_{CL} } \\ {\Delta tp_{QTZ} } & {\Delta tp_{CA} } & {\Delta tp_{CL} } \\ \end{array} } \right]\left[ {\begin{array}{*{20}c} {\alpha_{{^{QTZ} }} } \\ {\alpha_{CA} } \\ {\alpha_{CL} } \\ \end{array} } \right] = \left[ {\begin{array}{*{20}c} {\rho_{o} } \\ {Pe_{o} } \\ {\Delta tp_{o} } \\ \end{array} } \right], $$

with $\rho$, $Pe$ and $\Delta tp$ representing the density, the photoelectric factor and the compressional slowness of quartz [QTZ], calcite [CA] and clay [CL]. Also, there are inversion methods that consider a non-linear relationship between volume fractions and nuclear well logs^5,6^. However nuclear well logs such as potassium/uranium/thorium concentrations are often not available in practice.

The micromechanics SC approach proposed here intends to estimate the mineral volume fractions of terrigenous formations solely from downhole elastic measurements where the relationship of the observed physical elastic properties to the mineral volume concentrations is assumed to be non-linear. The SC scheme has the advantage that mineral volume fractions are obtained from density and slowness well logs, which are commonly available.

Micromechanics attempts to estimate the physical properties of rock composites from the physical properties and the geometric characteristics of their forming microstructures^7–10^. In particular, SC micromechanics method^11,12^ idealizes the rock composite as being a heterogeneous isotropic material containing a background isotropic matrix embedded with $n$ randomly distributed isotropic spherical inclusions of different types^13–17^. Here, the composite represents the terrigenous rock formation where either the matrix or the mineral inclusions can be represented by any of the following forming minerals: quartz, calcite and clay. Thus, for example, if quartz represents the matrix then calcite and clay are the inclusions. Also, if calcite is the matrix then quartz and clay are the inclusions. Therefore, calcite and quartz are inclusions when clay is the matrix. The system of non-linear SC micromechanics equations proposed in this contribution attempt to model the following rock elastic physical properties: the density $\rho_{o}$ and both the compressional $Vp_{o}$ and the shear $Vs_{o}$ velocities; where the velocities are calculated from compressional $\Delta tp_{o}$ and shear $\Delta ts_{o}$ slowness well logs. These SC equations are defined in terms of the unknown rock mineral volume fractions $\alpha$ and the known physical properties of the composing pure main minerals. Next, an objective function $F$ is built to compare the resulting modelled physical properties $\rho_{m}$, $Vp_{m}$ and $Vs_{m}$ to the observed physical properties $\rho_{o}$, $Vp_{o}$ and $Vs_{o}$. Then a gradient descent algorithm is used to optimize $F$, i.e., $F$ seeks for those mineral volume fractions $\alpha$ for which the difference between modelled and observed physical properties is minimum. Obtained volume fractions for calcite, clay and quartz are then compared to those from the LLSI method. Further, clay volume fractions from the SC method are also compared to results from GR linear method^18^. GR linear method is widely used as a guideline for clay volume fraction estimation since in this method clay volume fractions are estimated from direct gamma ray well log measurements on the borehole. Similar methods to estimate volume fractions for quartz and calcite from direct measurements on boreholes are not available in the literature. However, the quartz + calcite volume fraction assemblage can be easily calculated from GR linear method and therefore compared to quartz + calcite volume fraction assemblage from SC. In the next section SC method and gradient descent optimization algorithm are briefly presented followed by the presentation of the results.

## Methods

### Self-consistent method

In the SC method, the bulk modulus $k_{m}$, the shear modulus $\mu_{m}$ and the density $\rho_{m}$ of composites containing an isotropic matrix with $n$ randomly embedded inclusions can be estimated as follows^7^:

4 $$ k_{m} \left( \alpha \right) = k_{n + 1} + \mathop \sum \limits_{r = 1}^{n} \frac{{\alpha_{r} \left( {k_{r} - k_{n + 1} } \right)}}{{1 + 3\left( {k_{r} - k_{m} } \right)/(3k_{m} + 4\mu_{m} )}}\,, $$

5 $$ \mu_{m} \left( \alpha \right) = \mu_{n + 1} + \mathop \sum \limits_{r = 1}^{n} \frac{{\alpha_{r} \left( {\mu_{r} - \mu_{n + 1} } \right)}}{{1 + 6\left( {\mu_{r} - \mu_{m} } \right)\left( {k_{e} + 2\mu_{m} } \right)/\left[ {5\mu_{m} \left( {3k_{m} + 4\mu_{m} } \right)} \right]}}\,, $$

and

6 $$ \rho_{m} \left( \alpha \right) = \rho_{n + 1} + \mathop \sum \limits_{r = 1}^{n} \alpha_{r} \left( {\rho_{r} - \rho_{n + 1} } \right)\,, $$

where $k_{n + 1}$, $\mu_{n + 1}$ and $\rho_{n + 1}$ correspond to the bulk modulus, the shear modulus and the density of the mineral that represent the matrix. $k_{r}$, $\mu_{r}$ and $\rho_{r}$ are the bulk modulus, the shear modulus and density of the $r$th inclusion while $\alpha_{r}$ represents the volume fraction for each inclusion such that $\sum\nolimits_{r = 1}^{n + 1} {\alpha_{r} = 1}$. Modelled compressional and shear wave velocities $Vp_{m}$ and $Vs_{m}$ can then be expressed as^19^:

7 $$ Vp_{m} \left( \alpha \right) = \left( {\frac{{k_{m} \left( \alpha \right) + {4 \mathord{\left/ {\vphantom {4 {3\mu_{m} \left( \alpha \right)}}} \right. \kern-\nulldelimiterspace} {3\mu_{m} \left( \alpha \right)}}}}{{\rho_{m} \left( \alpha \right)}}} \right)^{{{1 \mathord{\left/ {\vphantom {1 2}} \right. \kern-\nulldelimiterspace} 2}}} $$

and

8 $$ Vs_{m} \left( \alpha \right) = \left( {\frac{{\mu_{m} \left( \alpha \right)}}{{\rho_{m} \left( \alpha \right)}}} \right)^{{{1 \mathord{\left/ {\vphantom {1 2}} \right. \kern-\nulldelimiterspace} 2}}} . $$

The solution of the non-linear Eqs. (4) and (5) must be found numerically. A detailed methodology to solve this system of equations can be found in Valdiviezo-Mijangos^20^ and Lizcano-Hernández, et al.^15^.

### Gradient descent optimization equations

Equations (4)–(8) are used to define the objective function $F$ that accounts, depth by depth, for the difference between the modelled and the data obtained from well logs:

9 $$ F\left( {\alpha ,\delta } \right) = \left( {\frac{{Vp_{m} \left( \alpha \right) - Vp_{o} }}{{\sigma_{Vp} }}} \right)^{2} + \left( {\frac{{Vs_{m} \left( \alpha \right) - Vs_{o} }}{{\sigma_{Vs} }}} \right)^{2} + \left( {\frac{{\rho_{m} \left( \alpha \right) - \rho_{o} \,\delta }}{{\sigma_{\rho } }}} \right)^{2} , $$

where $Vp_{o}$ and $Vs_{o}$ are the compressional and shear velocities obtained from sonic well logs. In Eq. (9) well log density $\rho_{o}$ is adjusted by a factor $\delta$ to assist to achieve convergence when minimizing $F$. On the other hand, $\sigma_{Vp}$, $\sigma_{Vs}$ and $\sigma_{\rho }$ represent the standard deviation associated to $Vp_{o}$, $Vs_{o}$ and $\rho_{o}$ respectively. Minimizing $F\left( {\alpha ,\delta } \right)$ with respect to $\alpha$ and $\delta$ by using the gradient descent method the following iterative equations are obtained^21^:

10 $$ \alpha_{r,j + 1} = \alpha_{r,j} - \frac{{\partial F_{j} }}{{\partial \alpha_{r} }}\frac{M}{{\left| {\nabla F_{j} } \right|}} $$

and

11 $$ \delta_{j + 1} = \delta_{j} - \frac{{\partial F_{j} }}{\partial \delta }\frac{M}{{\left| {\nabla F_{j} } \right|}}, $$

where $j$ is the number of iterations. Equations (9)–(11) allow then to estimate the mineral volume fraction values that attempt to best describe the observed well log data of the terrigenous formation.

### Gradient descent solution

**Figure 1:**
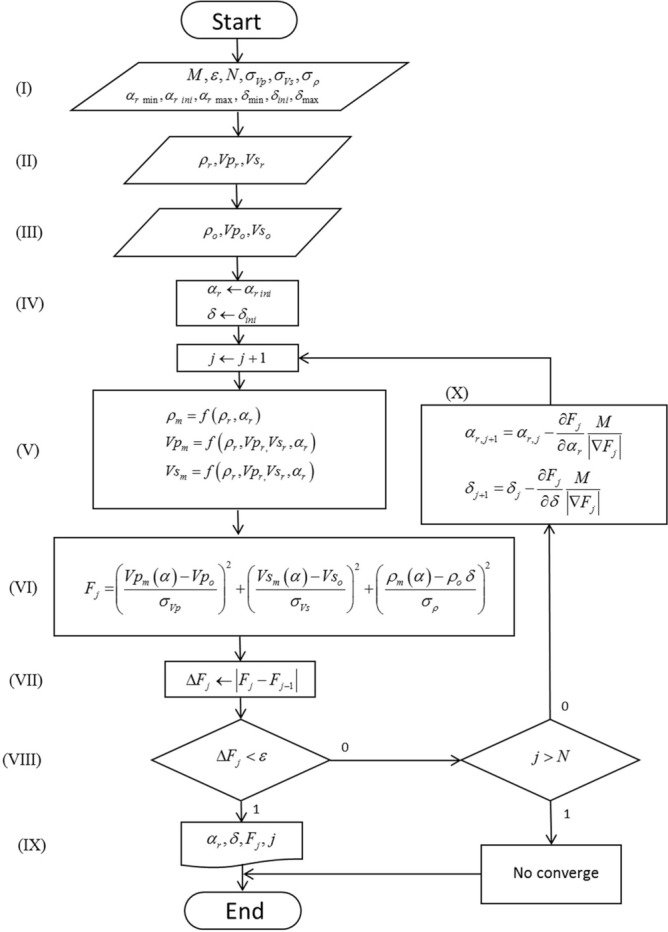
Gradient descent-based optimization algorithm flowchart.

The optimization programming flowchart used to estimate mineral volume is shown in Fig. 1. The main processes involving the optimization algorithm are as follows:

(I). Setting the initial constants and parameters such as: the convergence factor $M$, the tolerance $\varepsilon$, the maximum number of iterations $N$, the standard deviations $\sigma$ associated to the measured data and the initial, the minimum and the maximum range of values for both the volume fractions $\alpha$ and the density adjustment factor $\delta$.
(I). Selecting the input physical properties of each mineral: density $\rho_{r}$ and compressional and shear velocities $Vp_{r}$ and $Vs_{r}$
(II). Choosing of the physical properties that correspond to the observed data:$\rho_{o}$, $Vp_{o}$ and $Vs_{o}$.
(III). In this step, initial values of volume fractions $\alpha$ and the density adjustment factor $\delta$ are assigned.
(IV). Then, the physical properties and parameters defined in step (II) and (IV) are used to solve the system of self-consistent Eqs. (4) and (5). As a result, the first approximation of rock´s physical properties is obtained.
(V). The objective function $F\left( {\alpha ,\delta } \right)$ is then estimated
(VI). $\Delta F_{j}$ is estimated by computing the difference between $F_{j}$ and the previous iteration $F_{j - 1}$.
(VII). If $\Delta F_{j}$ is less than the given tolerance $\varepsilon$, the algorithm is stopped leading to step (IX). Otherwise, the following is verified: if $j > N$ the algorithm is stopped and convergence is not reached. If $j < N$ the algorithm continues to step (IX) where the gradient descent method is applied.

## Results and discussion: application to a terrigenous formation

**Figure 2:**
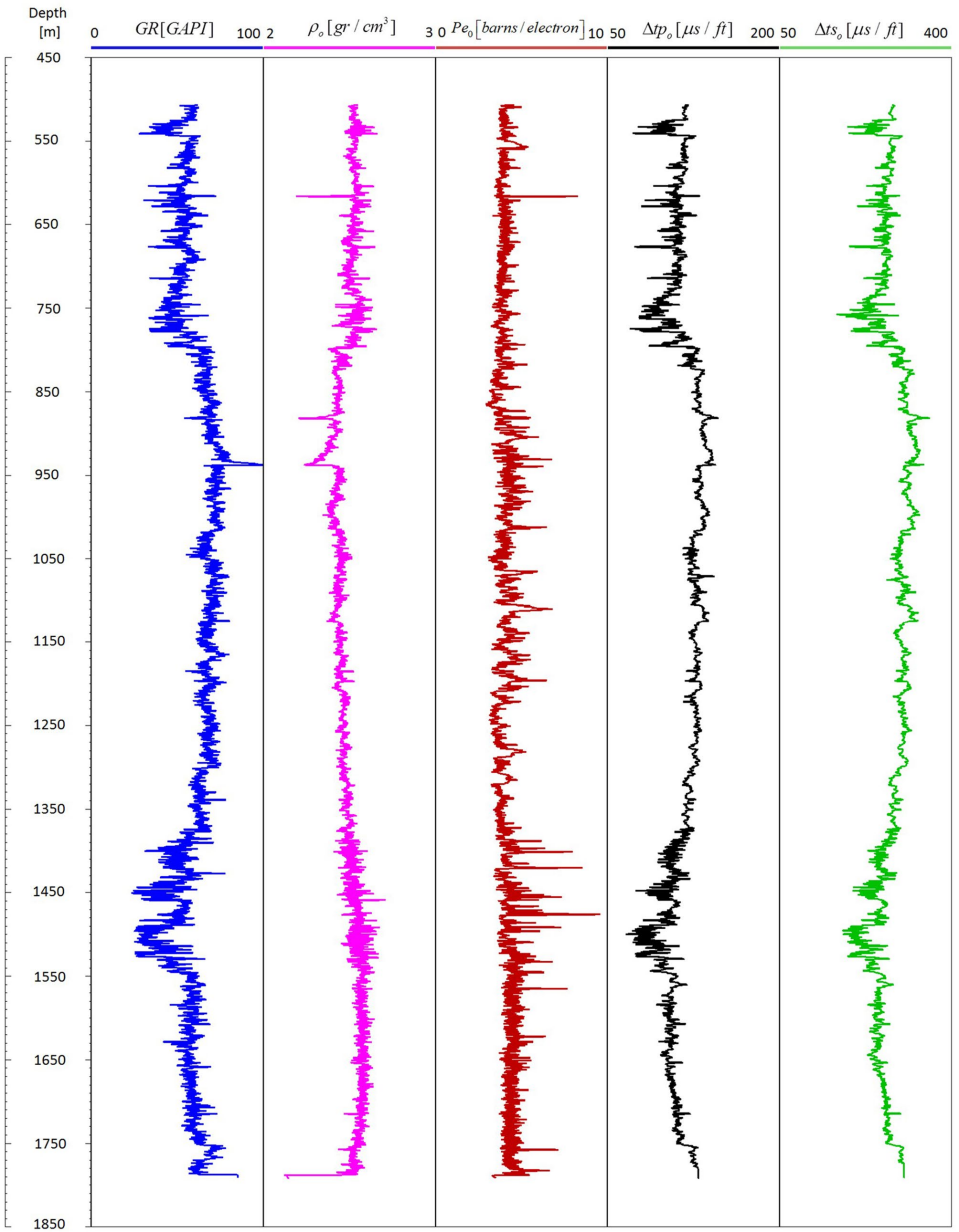
Well log data used as input.

Typical physical properties of the pure main minerals and well logs used as input data are shown in Fig. 2 and Table 1 respectively. Data in Fig. 2 went through a rigorous quality control process to account for systematic and random errors. This quality control process intends to guarantee the accuracy and precision of the well log data. Systematic errors can occur as a consequence of poor tool calibration, complex borehole environmental conditions and depth shifts between well loggings runs, to mention a few. These systematic errors are identified and removed using normalization techniques on the raw well log data. On the other hand, random errors arise from the precision limitations of the well logging tools. As such, when multiple measurements of the same type are taken at a given depth with the same tool, the measured values are reported to be shifted both lower and higher. However, these random errors are reduced towards zero by averaging a sufficient number of measurements.

**Table 1:** Physical properties of composing main minerals.

Mineral	$\rho$ (g/cm^3^)	*Pe* (barns/electron)	$Vp$ (km/s)	$Vs$ (km/s)
Clay	2.60	3.03	3.40	1.68
Quartz	2.65	1.82	6.00	4.09
Calcite	2.71	5.09	6.60	3.40

**Figure 3:**
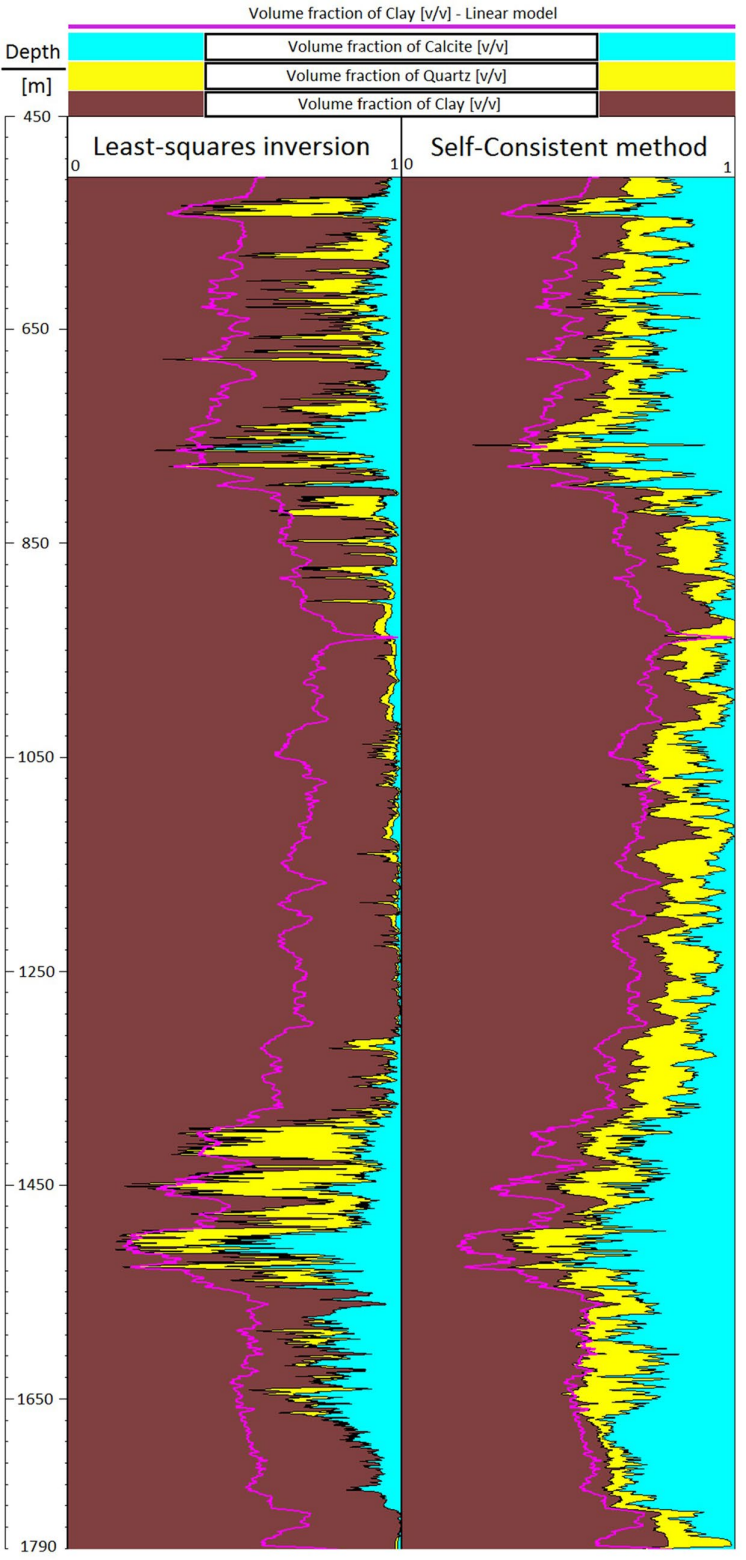
Depth mineral volume fractions profile computed by LLSI method [left] and SC method [right]. Clay volume fractions calculated from Eq. (12) are also shown. Observe that mineral volume fractions from the LLSI, SC and GR methods follow the same general trend.

**Figure 4:**
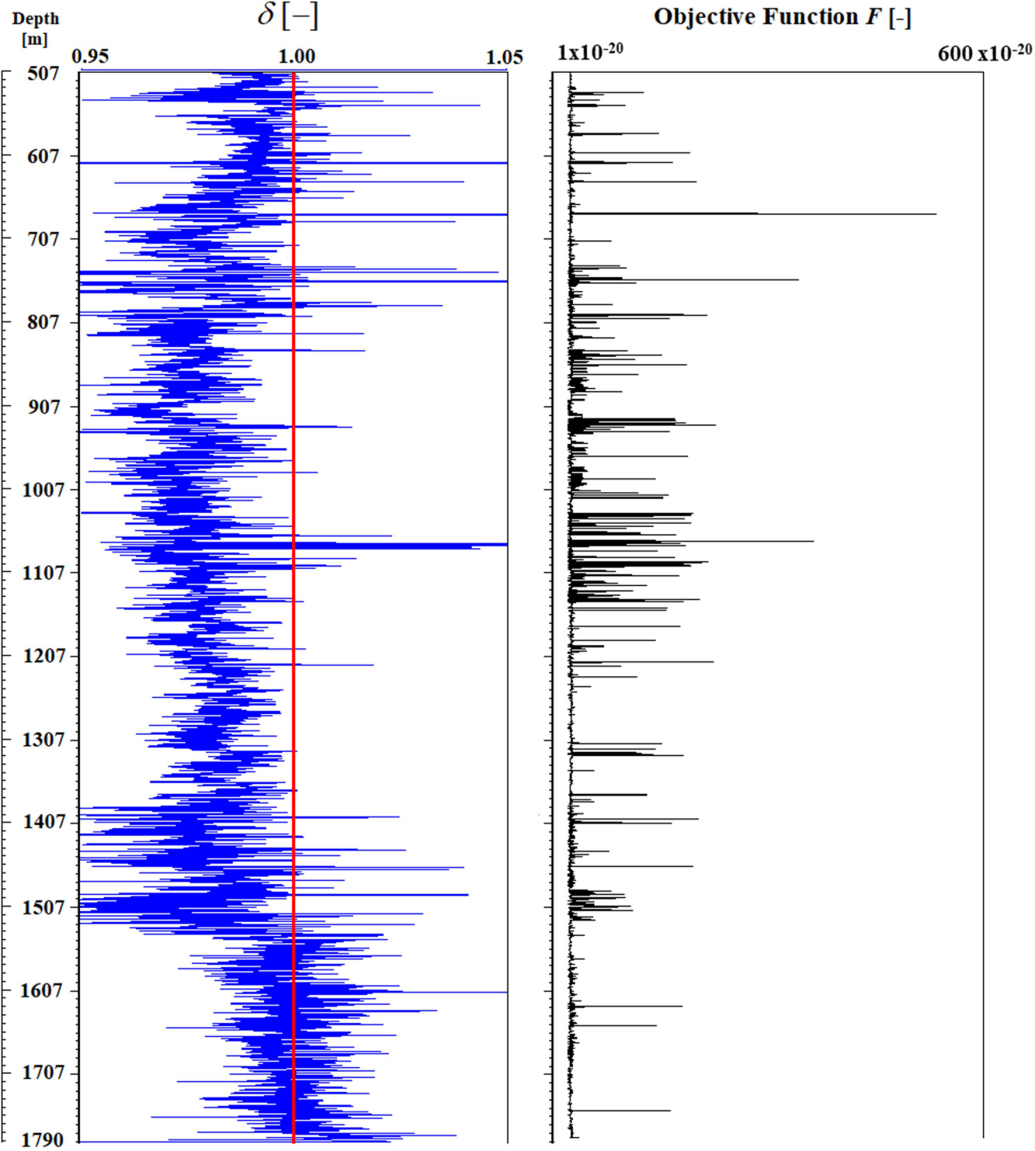
For each depth, density adjustment factor $\delta$ [left] and resulting final objective F [right] upon achieving convergence after $N$ iterations when using the SC method. A value of $\delta = 1$ [red line] means that the observed density does not need to be adjusted to achieve convergence when minimizing *F* to compute $\alpha$.

**Figure 5:**
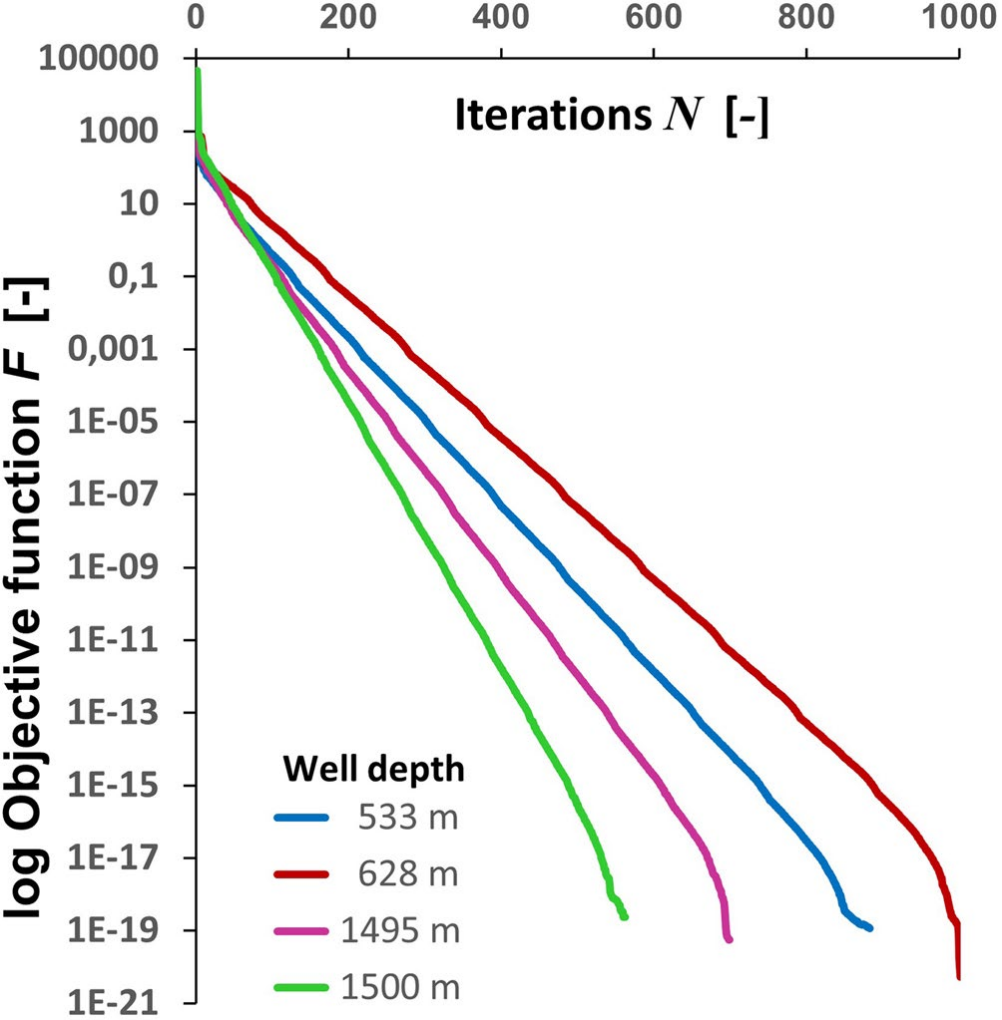
Examples of convergence of the objective function *F* after *N* iterations at four different depths.

Gamma ray GR well log in Fig. 2 is used to generate a guideline for volume clay estimation $\alpha_{CL\,[GR]}$ as follows^18^:

12 $$ \alpha_{CL\,[GR]} = \frac{{GR - GR_{\min } }}{{GR_{\max } - \,GR_{\min } }}\,, $$

where $GR_{\min }$ and $GR_{\max }$ correspond to the gamma ray values of clean sandstones and shale formations respectively. Equation (12), known as the gamma ray linear equation, is widely used in petrophysical analysis to directly obtain clay volume concentrations. Figure 3 illustrates the clay volume estimation using the GR linear model [with $GR_{\min } = 20$ GAPI and $GR_{\max } = 90$ GAPI] along with the mineralogy computed by LLSI and SC methods. Note that computed mineralogy from LLSI method follows the same general trend as that computed by using the SC method. Clay volume fraction estimated by LLSI and SC methods also exhibits the same trend as the GR linear model. Figure 4 shows, for each depth, both the density adjustment factor $\delta$ used and the final objective function *F* that results from computing the mineral volume fractions $\alpha$ using the SC method (Fig. 3, right). Observe that the gradient descent-based optimization algorithm pictured in Fig. 1 can estimate mineral volume fractions such that the final values of *F* are of the order of 10^–20^. Examples of the convergence of *F* against the number of iterations *N* at four different depths are given in Fig. 5.

**Figure 6:**
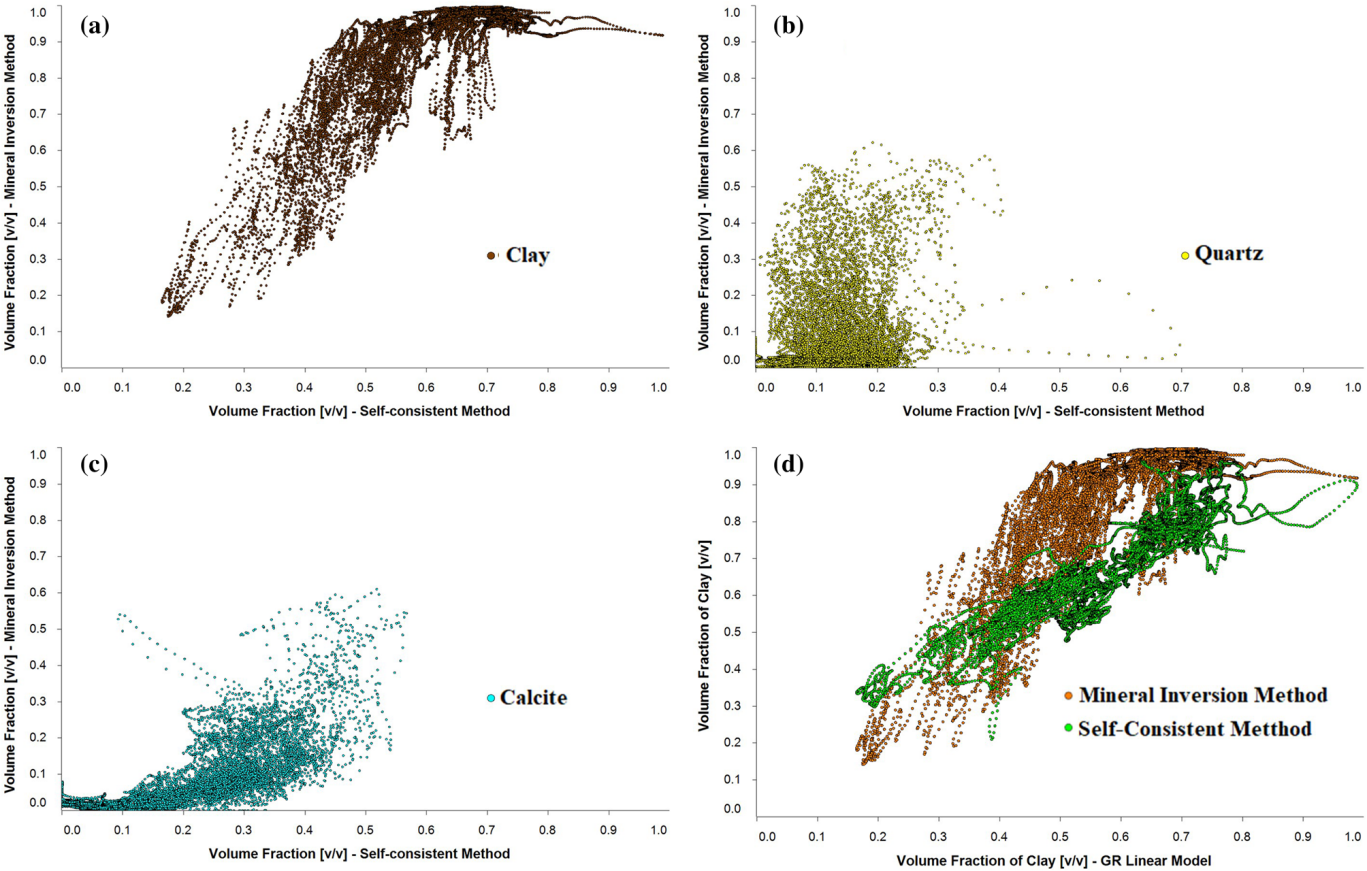
Volume fraction comparative. Cross plots of the computed volume fractions of clay (**a**), quartz (**b**) and calcite (**c**) using SC and LLSI methods. (**d**) Clay volume fraction obtained from SC and LLSI compared to GR linear model. Note that computed quartz volume fractions from SC method are highly dispersed from those of LLSI method.

Figure 6a–c show a cross plot of volume fraction of clay, quartz and calcite obtained from LLSI and SC methods. In Fig. 6d, clay volume fractions computed from LLSI and SC methods are compared to GR linear model. Table 2 shows the Pearson coefficient of correlation P^22^ for the curves portrayed in Fig. 6a–d.

**Table 2:** Pearson coefficient of correlation between depth mineral volume fractions profiles estimated by LLSI, SC and GR methods.

	SC				LLSI	
	Clay	Quartz	Calcite	Quartz + Calcite	Clay	Quartz + Calcite
**LLSI**						
Clay	0.79				1	
Quartz		0.20				
Calcite			0.82			
Quartz + Calcite				0.82		1
**GR**						
Clay	0.83				0.77	
Quartz + Calcite				0.83		0.78

Thus, while curves for clay and calcite are highly correlated with Pearson´s correlation coefficients of P = 0.79 and P = 0.82 respectively, the correlation between curves for quartz is negligible with P = 0.20. However, the total volume fraction that results from adding the volume fractions of quartz with volume fractions of calcite in both methods also exhibits a high correlation value of P = 0.82.

On the other hand, the Pearson coefficient of clay concentrations from GR linear model with LLSI and SC method is 0.83 in both cases. Further, since equivalent relationships to Eq. (12) for quartz and calcite are not available in the literature, the total volume fractions of quartz and calcite from the GR well log can be easily estimated as follows: $\alpha_{{\left( {QTZ + CA} \right)\,\left[ {GR} \right]}} = 1 - \alpha_{CL\,[GR]}$. The latter leads to values of P = 0.78 and P = 0.83 when correlating results from GR linear model with SC and LLSI methods respectively.

These results suggest that SC method is best suited to estimate clay and calcite volume fractions as well as volume fractions of quartz + calcite mineral assemblages rather than estimating quartz volume fractions. The fact that SC method is not giving good correlation with LLSI method for quartz is because the photoelectric effect well log is more sensitive than elastic well logs to indicate the presence of quartz minerals contained in the rock formation. Nevertheless, the SC method was able to indicate the general distribution of quartz. Also, note that SC modelling computes mineral volume fractions only from density and elastic sonic well logs. This is particularly helpful in the case where $Pe_{o}$ and GR well log measurements are either not available or incomplete along the borehole.

## Conclusions

A non-linear micromechanics SC method to calculate clay, calcite and quartz volume fractions from well logs obtained on a terrigenous formation have been implemented. The latter is achieved by using a novel algorithm to solve a gradient descent-based optimization algorithm focused on computing rock mineralogy from density and elastic sonic well logs. The best results, when compared with conventional linear inversion and GR linear methods,

are obtained for clay and calcite mineral concentrations and calcite + quartz mineral volume fractions assemblages. However, the main advantage of the SC method is that mineral volume fractions can be calculated solely from the modelled elastic response of the formation. As such, mineral concentrations obtained from the SC method also have the potential to be scaled to regional seismic scales where $Pe_{o}$ and GR data are not available.
